# Supplementary material for: Postpartum hemorrhage in Suriname: A national descriptive study of hospital births and an audit of case management
Source: PLoS One. 2020 Dec 18;15(12):e0244087. doi: 10.1371/journal.pone.0244087 (PMC7748130; doi:10.1371/journal.pone.0244087)
Supplement: S1 File — (DOCX) [file pone.0244087.s001.docx]

**S1 Fig. Categories of available maternal characteristics, pregnancy and delivery outcomes based on international classification**

**
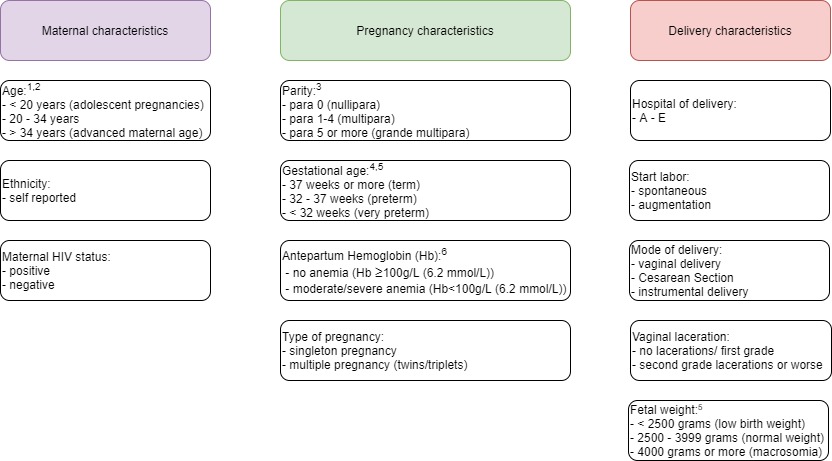
**

References:

1. Kahveci, B., Melekoglu, R., Evruke, I. C. & Cetin, C. The effect of advanced maternal age on perinatal outcomes in nulliparous singleton pregnancies. *BMC Pregnancy Childbirth* **18**, 1–7 (2018).

2. WHO factsheet Adolescent pregnancies. (2020). Available at: https://www.who.int/news-room/fact-sheets/detail/adolescent-pregnancy. (Accessed: 19th March 2020)

3. Muniro, Z., Tarimo, C. S., Mahande, M. J., Maro, E. & McHome, B. Grand multiparity as a predictor of adverse pregnancy outcome among women who delivered at a tertiary hospital in Northern Tanzania. *BMC Pregnancy Childbirth* **19**, (2019).

4. Goldenberg, R. L., Culhane, J. F., Iams, J. D. & Romero, R. Epidemiology and causes of preterm birth. *Lancet* **371**, 75–84 (2008).

5. ICD-10 Version:2019. Available at: https://icd.who.int/browse10/2019/en#/XVI. (Accessed: 19th March 2020)

6. WHO. Haemoglobin concentrations for the diagnosis of anaemia and assessment of severity. 1–6 (2011). doi:2011

**S1 Table**. **Maternal and perinatal characteristics of the hospital deliveries in 2017 in Suriname**

| **Hospital** | **A** | **B** | **C** | **D** | | **E** | | |
| --- | --- | --- | --- | --- | --- | --- | --- | --- |
| Total n (%) | 2101 (100%) | 2654 (100%) | 367 (100%) | 2456 (100%) | | 1493 (100%) | | |
| Live births | 2018 (96.0) | 2616(98.6) | 361 (98.4) | 2416 (98.4) | | 1478 (99.0) | | |
| Stillbirths | 83 (4.0) | 38 (1.4) | 6 (1.6) | 40 (1.6) | | 15 (1.0) | | |
| **Maternal Characteristics** | | | | | | | |  |
| **Age (years)** | | | | |  | |  |  |
| 12 – 19 | 382 (18.2) | 355 (13.4) | 67 (18.3) | 384 (15.7) | | 101(6.8) | | |
| 20 - 34 | 1465 (69.9) | 1851 (69.8) | 264 (71.9) | 1724 (70.4) | | 1170 (78.4) | | |
| > 35 | 250 (11.9) | 447 (16.8) | 36 (9.8) | 342 (14) | | 221 (14.8) | | |
| *Missing* | 4 | 1 | 0 | 6 | | 1 | | |
| **Ethnicity** | | | | |  | |  |  |
| Maroon | 863 (41.8) | 785 (29.6) | 2 (0.6) | 844 (34.4) | | 90 (6.1) | | |
| Creole | 541 (26.2) | 589 (22.2) | 18 (5.2) | 517 (21.1) | | 305 (20.5) | | |
| Hindustani | 328 (15.9) | 477 (18.0) | 196 (56.6) | 382 (15.6) | | 272 (18.3) | | |
| Other^1^ | 155 (7.5) | 381 (14.4) | 66 (19.1) | 394 (16.0) | | 498 (33.5) | | |
| Javanese | 102 (4.9) | 324 (12.2) | 40 (11.6) | 198 (8.1) | | 290 (19.5) | | |
| Indigenous | 74 (3.6) | 93 (3.5) | 24 (6.9) | 120 (4.9) | | 31 (2.1) | | |
| *Missing* | *38* | *5* | *21* | *1* | | *7* | | |
| **Maternal HIV status** | | | | | | | |  |
| Positive | 39 (1.9) | 14 (0.5) | N/A | 1 (0) | | 14 (0.9) | | |
| *Missing* | *1* | *0* |  | *1* | | *0* | | |
| **Pregnancy characteristics** | | | | | | | |  |
| **Parity** | | | | |  | |  |  |
| 0 | 601 (28.7) | 1000 (38.0) | 147 (40.2) | 721 (29.4) | | 643 (43.1) | | |
| 1 – 4 | 1265 (60.4) | 1423 (54.0) | 206 (56.3) | 1495 (60.9) | | 823 (55.2) | | |
| > 5 | 227 (10.8) | 210 (8.0) | 13 (3.6) | 240 (9.8) | | 25 (1.7) | | |
| *Missing* | *8* | *21* | *1* | *0* | | *2* | | |
| **Antepartum anemia** | | | | |  | |  |  |
| Anemia^2^ | 731 (50.4) | N/A | N/A | N/A | | 347 (24.2) | | |
| *Missing* | *652* |  |  |  | | *58* | | |
| **Type of pregnancy** | | | | |  | |  |  |
| Multiple pregnancy | 30 (1.4) | 41 (1.5) | 1 (0.3) | 29 (1.2) | | 15 (1) | | |
| **Delivery characteristics** | | | | | | | |  |
| **Onset of labor** | | | | | | | |  |
| Augmentation | 781 (37.2) | 781 (37.2) | 101 (27.5) | N/A | | 445 (34.2) | | |
| *Missing* | *1* | *2054* | *266* |  | | *193* | | |
| **Mode of delivery** | | | | |  | |  |  |
| Spontaneous | 1725 (82.1) | 2026 (76.3) | 285 (77.7) | 1827 (74.4) | | 927 (62.1) | | |
| Caesarean Section | 364 (17.3) | 552 (20.8) | 53 (14.4) | 604 (24.6) | | 564 (37.8) | | |
| Instrumental | 12 (0.6) | 76 (2.9) | 29 (7.9) | 25 (1.0) | | 2 (0.1) | | |
| **AMTSL ^3^**  **only for severe PPH n=155 (100%)** |  |  |  |  | |  | | |
| Oxytocin prevention applied | 19 (67.9) | 30 (75.0) | 7 (77.8) | 29 (46.0) | | 11 (73.3) | | |
| *Missing* | *16* | *13* | *1* | *31* | | *4* | | |
| **Vaginal laceration** | | | | | | | |  |
| 2^nd^ grade or higher | 434 (27.8) | 771 (72.7) | 101 (95.3) | N/A | | 469 (58.6) | | |
| *Missing* | 541 | 1593 | 261 |  | | 1692 | | |
| **Birthweight (grams)** | | | | | | | |  |
| < 2500 | **435 (20.9)** | 318 (12.0) | 55 (15.1) | 354 (14.4) | | 164 (11.0) | | |
| 2500 – 3999 | 1599 (76.7) | 2234 (84.6) | 293 (80.3) | 2054 (83.7) | | 1258 (84.6) | | |
| > 4000 | 52 (2.5) | 88 (3.3) | **17 (4.7)** | 46 (1.9) | | 65 (4.4) | | |
| *Missing* | *15* | *14* | *2* | *2* | | *6* | | |
| **Legend**  ^1^ Ethnicity other: Mixed, Chinese, Brazilian, Caucasian, unknown  ^2^ Hemoglobin < 100 g/l or 6.1 mmol/l  ^3^ AMTSL: Active Management of the Third Stage of Labor  N/A = not available | | | | | | | |  |

**S2 Fig. Availability of data on causes and management of postpartum hemorrhage (PPH) to perform a criteria-based audit**
